# Evaluation of 6 candidate genes on chromosome 11q23 for coeliac disease susceptibility: a case control study

**DOI:** 10.1186/1471-2350-11-76

**Published:** 2010-05-17

**Authors:** Karen Brophy, Anthony W Ryan, Graham Turner, Valerie Trimble, Kunal D Patel, Colm O'Morain, Nicholas P Kennedy, Brian Egan, Eimear Close, Garrett Lawlor, Padraic MacMathuna, Fiona M Stevens, Mohamed Abuzakouk, Conleth Feighery, Dermot Kelleher, Ross McManus

**Affiliations:** 1 Department of Clinical Medicine, Trinity Centre for Health Sciences, Trinity College Dublin, St James's Hospital, Dublin, Ireland; 2 Institute of Molecular Medicine, Trinity Centre for Health Sciences, St James's Hospital, Dublin, Ireland; 3 Department of Immunology, Trinity College, Trinity Centre for Health Sciences, St James's Hospital, Dublin, Ireland; 4 Department of Clinical Medicine, Trinity College, Trinity Centre for Health Sciences, The Adelaide & Meath Hospital Dublin, Incorporating The National Children's Hospital, Tallaght, Dublin, Ireland; 5 Gastrointestinal Unit, Mater Misericordiae University Hospital, Dublin, Ireland; 6 Department of Medicine, National University of Ireland, Galway, Ireland; 7 Department of Immunology, Hull Royal Infirmary, Hull, UK

## Abstract

**Background:**

Recent whole genome analysis and follow-up studies have identified many new risk variants for coeliac disease (CD, gluten intolerance). The majority of newly associated regions encode candidate genes with a clear functional role in T-cell regulation. Furthermore, the newly discovered risk loci, together with the well established HLA locus, account for less than 50% of the heritability of CD, suggesting that numerous additional loci remain undiscovered. Linkage studies have identified some well-replicated risk regions, most notably chromosome 5q31 and 11q23.

**Methods:**

We have evaluated six candidate genes in one of these regions (11q23), namely *CD3E*, *CD3D*, *CD3G*, *IL10RA*, *THY1 *and *IL18*, as risk factors for CD using a 2-phase candidate gene approach directed at chromosome 11q. 377 CD cases and 349 ethnically matched controls were used in the initial screening, followed by an extended sample of 171 additional coeliac cases and 536 additional controls.

**Results:**

Promotor SNPs (*-607, -137*) in the *IL18 *gene, which has shown association with several autoimmune diseases, initially suggested association with CD (P < 0.05). Follow-up analyses of an extended sample supported the same, moderate effect (P < 0.05) for one of these. Haplotype analysis of *IL18-137/-607 *also supported this effect, primarily due to one relatively rare haplotype *IL18-607C/-137C *(P < 0.0001), which was independently associated in two case-control comparisons. This same haplotype has been noted in rheumatoid arthritis.

**Conclusion:**

Haplotypes of the *IL18 *promotor region may contribute to CD risk, consistent with this cytokine's role in maintaining inflammation in active CD.

## Background

Coeliac disease (CD), or gluten intolerance is an autoimmune inflammatory condition of the small bowel, precipitated by gluten and related proteins from dietary grains such as wheat, barley and rye. Removal of these proteins from the diet is usually sufficient for complete remission of symptoms. The primary genetic determinant of CD risk is inheritance of the HLA-DQ2 molecule, encoded by genes (*HLADQA1 *and *HLADQB1*) on chromosome 6p21. However, other genetic risk factors are known to exist, a number of which have been identified by linkage studies [1-3] or, more recently, whole genome SNP analyses [4,5].

Several genomic regions thought to harbour susceptibility genes have been identified by family studies. Linkage of several of these regions to CD has been replicated in independent studies from multiple populations. Of these, chromosomal regions 5q31 and 11q23 have been most successfully replicated. The linkage of 11q markers to coeliac disease have been demonstrated by 3 studies [1-3]. Markers from 115.8 Mb (D11S4111) to 123.6 (D11S4464) have shown linkage, with peak associations at D11S4142 at chromosome position 115.3 Mb [2], and D11S4464 at 123.6 Mb [3].

This region harbours several candidate genes for CD susceptibility. Louka et al [6] reported no association between CD and functional polymorphisms in the *MMP1 *and *MMP3 *genes. However, Mora et al [7] reported a sex specific association between an *MMP3 *promotor polymorphism and coeliac disease.

The *CD3 *genes (118.2 Mb), *CD3*-*epsilon *(*CD3E*), *CD3*-*delta *(*CD3D*) and *CD3*-*gamma *(*CD3G*), lie within 50 kb of each other in this region, forming part of the T-cell-receptor (TCR) complex. This complex consists of either alpha and beta or gamma and delta variant chains, in association with the invariant chains *CD3E*, *CD3D*, *CD3G *and *CD3*-zeta (*CD3Z*). During development, this *CD3 *protein complex plays an important role in the transition of thymocytes from immature precursors to the final mature CD4+ or CD8+ single-positive T-cell. Studies have shown that the *CD3 *components are essential during the early stages of human thymopoiesis and deficiencies in these genes have been linked with severe combined immunodeficiency [8,9]. One preliminary study has been carried out to date on the association between *CD3 *and autoimmune disease [10], which identified a significant association between *CD3D *and type 1 diabetes with the use of microsatellites.

Also located on 11q23 is the *THY1 *gene (119.2 Mb). This gene encodes a major cell surface glycoprotein characteristic of T-cells and is a member of the immunoglobulin supergene family. Although the role of this protein is not fully elucidated, its position as a cell surface molecule on T-cells and its involvement in cell-cell interactions [11] make it a candidate gene for coeliac disease pathogenesis.

The *IL10RA *gene (117.9 Mb) encodes the interleukin 10 receptor-alpha chain of the IL-10 receptor complex. This molecule is the receptor for *IL10*, an anti-inflammatory cytokine produced by a subset of activated T-cells, B-cells and macrophages, which has a role in a variety of immunoregulatory functions [12]. Genetic variants of *IL10RA *have been shown to inhibit the production of TNF-alpha [13], a protein known to play a role in coeliac disease. Coeliac patients in remission produce significantly higher levels of TNFa than controls and while this appears to be a genetically inherited trait, it does not appear to be due solely to genetic variation at the *TNF *locus[14] [Daly and McManus, unpublished observation]. Thus, polymorphisms at other loci which could alter TNF production are high priorities for investigation as candidate genes.

A polymorphism in *IL10 *(-1087) has been linked to a number of autoimmune diseases including inflammatory bowel disease, rheumatoid arthritis (RA) and systemic lupus erythematosus [15]. A significant association between the *IL10-1087 *polymorphism and coeliac disease has been shown [16]. However, this association failed to be replicated in subsequent work [17]. The *IL10-1087 *polymorphism has also been shown to be associated with differing levels of IgA anti-endomyseal and anti-tissue transglutaminase antibodies in CD patients [16], while recombinant human interleukin 10 has been shown to suppress gliadin dependent T-cell activation in *ex vivo *cultured coeliac intestinal mucosa [18]. These observations suggest that *IL10 *may be a factor in the pathogenesis of CD. However, as *IL10 *signals through the specific receptor, *IL10RA*, a possible role for this receptor molecule in disease, mediated through the *IL10 *pathway, should also be considered. The strength of *IL10RA *as a candidate gene is enhanced by its physical proximity to D11S976, a microsatellite marker which has been highlighted in a number of family studies as showing linkage with CD.

Also present as a candidate gene in this region is *IL18 *(112.0 Mb), also known as interferon-gamma-inducing factor. Though this gene, located at chromosomal position 11q22.2-22.3, is some distance from the major linkage peaks in the region, it may still fall within the region of linkage as it is located between a marker shown to be linked to disease (D11S4111) at position 115.8 MB and the next tested marker (D11S898) at 101.0 MB which was not linked to disease [2]. *IL18 *is a proinflamatory cytokine which, in synergy with IL12, promotes development of the Th1 lymphocyte response by induction of γ-interferon (IFN-γ). The latter is highly produced in CD lesions, and is known to play an important role in inflammatory and infectious diseases [19]. Furthermore, increased serum levels of *IL18 *have been identified in patients with autoimmune diseases such as RA [20] and acute asthma [21]. Two polymorphisms in the promoter region of the gene have shown evidence of altering *IL18 *protein expression. One polymorphism located at position *-607 *has been found to disrupt a potential cAMP-response element protein-binding site, while the other at position *-137 *alters a consensus H4TF-1 nuclear factor-binding site. Multiple sclerosis patients homozygous for the *-607*C and *-137*G alleles have higher levels of *IL18 *mRNA compared to other diplotypes, suggesting that these polymorphisms do indeed regulate activity of the gene [22]. More recent results point to a haplotypic effect based on other polymorphisms [23]. Numerous genetic association studies have been carried out on these SNPs and others in the gene, to investigate if any association exists with various autoimmune diseases. Significant association has been shown between *IL18 *and type 1 diabetes [24], Crohn's disease [25], atopic eczema [26], inflammatory bowel disease [27], and asthma [28]. Other studies have failed to find a disease association between *IL18 *and several diseases, among which are studies on coeliac disease [29], RA [30], type 1 diabetes [31-33], and periodontitis [34]. A recent study, which included analysis of the Irish samples analysed in this study, has identified functional genetic variants at *IL18RAP*, a receptor for IL18, as a risk factor in coeliac disease [35].

Here we report candidate gene analysis of the above genes, using a haplotype tagging approach to maximise coverage of the common genetic variants in European populations. In addition to the analyses performed for this study, we also tested for epistatic effects between *IL18 *and *IL18RAP*.

## Methods

377 biopsy-confirmed unrelated coeliac patients and 349 controls were used as an initial screening population. All controls were healthy, randomly selected blood donors, and all case and control subjects were of uniform Irish ancestry. Sample characteristics are summarised in Table 1. Informed consent was obtained from all study subjects and the study was approved by the Ethics Committees of St James's Hospital, Dublin and the Mater Misericordiae University Hospital, Dublin. 10% of samples were re-genotyped anonymously in order to evaluate error rates. SNPs with an uncorrected P value < 0.05 on the initial screen were then tested on an additional sample of 171 coeliacs and 536 controls and the data pooled. Coeliac samples are biopsy proven, the significant majority (> 80%) of which have the severe phenotype (Marsh III or greater) at the time of biopsy. In the absence of clear damage to the villous architecture, patients are diagnosed on the basis of an IEL infiltrate and or crypt hyperplasia (Marsh I/II) AND positivity for antibody tests (both anti-EMA and anti-tTG) and where possible, evidence of clinical improvement on a gluten free diet. *HLA-DQB1 *testing of approximately 300 patients shows over 99% are positive for one or more of the known *HLA-DQB1 *alleles, HLA-DQB1*0201/0202/0301/0302.

**Table 1:** Sample sizes, age and sex for cases and controls.

	N	% Female	Age (mean)	SD
Coeliac 1	377	71	50.3	14.2
Coeliac 2	171	68	44.5	15.5
Control 1	349	57	32.6	8.5
Control 2	536	69	35.6	12.4

In order to optimise genotyping efficiency, haplotype tags were identified for each gene. *IL10RA *and *IL18 *haplotype tags were identified from complete, or near complete, phased re-sequence data from *SeattleSNPs *http://pga.gs.washington.edu/education.html and *Innate Immunity*http://www.pharmgat.org/IIPGA2/index_html databases. Re-sequence data were not available for the 3 *CD3 *genes or *THY1*. For these, genotype data were downloaded from the NCBI and Perlegen databases and gametic phase was assigned. Haplotype tags for each of the above genes were identified using the SNPtagger program, in order to tag common haplotypes (> 5%) for SNPs with minor allele frequency > 0.05. Genotyping was performed using Amplifluor™ or Taqman™ technology. Tests for allele and haplotype frequency heterogeneity were performed using Hitagene, Genepop[36], Haplostats[37] and Phasev2 [38]. Tests for gene-gene interaction were performed using Plink[39].

## Results

All loci conformed to Hardy-Weinberg Equilibrium (P > 0.05) in all populations, with the exception of *CD3E *rs1945764 in control population 1 (P = 0.042). Anonymous genotype duplication suggested an error rate < 1%. Patterns of linkage disequilibrium (LD) are presented in Supplementary Figure S1 (Additional File 1). All polymorphic sites in *IL10RA*, *CD3D*, *CD3E*, *CD3G*, *THY1 *and *IL18 *were analysed for allelic, genotypic and carrier status association with disease individually and results are summarised in Table 2. No polymorphism within the *IL10RA*, *CD3D*, *CD3E*, *CD3G *or *THY1 *genes were found to be significantly associated with disease in this study. In the initial phase, two polymorphisms in the *IL18 *gene (*IL18-137 *rs187238 and *IL18-607 *rs1946518) showed a significant association with disease prior to correction for multiple testing. *IL18-137 *was significantly associated with disease (genotype frequency heterogeneity, P = 0.0380, Table 2). *IL18-607 *also showed a significant association with disease status (genotype frequency heterogeneity, P = 0.005; carrier status for the major allele, P = 0.001, Odds Ratio = 1.955 [CI, 1.30-2.95]).

**Table 2:** Comparison of genotypic frequencies (%) between Coeliac and Control samples (Table 1) and the pooled Coeliac (1 + 2) and Control (1 + 2) samples.

		A1A1	A1A2	A2A2	p
**Coeliac 1 Vs Control 1**					
IL10RA	Coeliac 1	86 (23.1)	192 (51.8)	93 (25.1)	
rs2256111 A/G	Control 1	69 (19.8)	192 (55.0)	88 (25.2)	0.514
IL10RA	Coeliac 1	255 (68.0)	101 (26.9)	19 (5.1)	
rs4252272 A/G	Control 1	226 (64.8)	115 (33.0)	8 (2.3)	0.068
IL10RA	Coeliac 1	171 (45.4)	169 (44.8)	37 (9.8)	
rs2229113 G/A	Control 1	150 (43.7)	167 (48.7)	26 (7.6)	0.426
IL10RA	Coeliac 1	113 (30.3)	191 (51.2)	69 (18.5)	
rs9610 G/A	Control 1	96 (28.0)	184 (53.6)	63 (18.4)	0.767
CD3E	Coeliac 1	118 (33.3)	178 (50.3)	58 (16.4)	
rs3782042 G/A	Control 1	133 (39.6)	158 (47.0)	45 (13.4)	0.196
CD3E	Coeliac 1	158 (49.7)	131 (41.2)	29 (9.1)	
rs1945764 T/C	Control 1	155 (45.9)	160 (47.3)	23 (6.8)	0.223
CD3DG	Coeliac 1	193 (54.4)	137 (38.6)	25 (7.0)	
rs2276423 G/C	Control 1	171 (49.9)	142 (41.4)	30 (8.8)	0.434
CD3DG	Coeliac 1	240 (66.5)	109 (30.2)	12 (3.3)	
rs3181259 G/A	Control 1	238 (69.6)	97 (28.4)	7 (2.1)	0.469
CD3DG	Coeliac 1	147 (41.9)	159 (45.3)	45 (12.8)	
rs1561966 G/A	Control 1	129 (38.2)	170 (50.3)	39 (11.5)	0.422
CD3DG	Coeliac 1	187 (52.5)	139 (39.0)	30 (8.4)	
rs7949185 T/C	Control 1	183 (53.2)	132 (38.4)	29 (8.4)	0.983
THY1	Coeliac 1	161 (43.5)	171 (46.2)	38 (10.3)	
rs1894006 G/A	Control 1	161 (47.1)	155 (45.3)	26 (7.6)	0.380
THY1	Coeliac 1	122 (34.3)	173 (48.6)	61 (17.1)	
rs1001205 G/A	Control 1	127 (37.9)	160 (47.8)	48 (14.3)	0.468
IL18	Coeliac 1	145 (44.2)	147 (44.8)	36 (11.0)	
rs2043055 A/G	Control 1	116 (38.2)	156 (51.3)	32 (10.5)	0.244
IL18	Coeliac 1	154 (50.3)	135 (44.1)	17 (5.6)	
* -137 rs187238 G/C	Control 1	184 (54.6)	121 (35.9)	32 (9.5)	0.038
IL18	Coeliac 1	227 (75.7)	68 (22.7)	5 (1.7)	
rs5744241 A/G	Control 1	237 (82.3)	49 (17.0)	2 (0.7)	0.114
IL18	Coeliac 1	138 (36.6)	192 (51.1)	46 (12.2)	
* -607 rs1946518 C/A	Control 1	100 (31.1)	153 (47.5)	69 (21.4)	0.005
					
**Coeliac 2 Vs Control 2**					
IL18	Coeliac 2	99 (57.9)	56 (32.7)	16 (9.4)	
-137 rs187238 G/C	Control 2	272 (52.7)	208 (40.2)	37 (7.1)	0.641
IL18	Coeliac 2	50 (42.4)	48 (40.7)	20 (16.9)	
-607 rs1946518 C/A	Control 2	201 (37.5)	256 (47.8)	79 (14.7)	0.712
					
**Coeliac (1 + 2) Vs Controls (1 + 2)**					
IL18	Coeliac	253 (53.0)	191 (40.0)	33 (6.9)	
-137 rs187238 G/C	Control	456 (53.4)	329 (38.5)	69 (8.1)	0.696
IL18	Coeliac	188 (38.1)	240 (48.6)	66 (13.4)	
-607 rs1946518 C/A	Control	301 (35.1)	409 (47.7)	148 (17.2)	0.042

P values (uncorrected for multiple testing) are for genotype heterogeneity (Genepop). SNPs with a reportedly functional role are marked with an asterisk (*).

These polymorphisms were further investigated in an augmented sample with 171 additional coeliacs and 536 random controls from the Irish Blood Transfusion Service (Table 2). These controls were compared with coeliacs separately and as a single pooled population, bringing the total number of control samples to 885. Comparison of the pooled coeliac sample with pooled control samples suggested a moderate effect for *IL18-607*, though it was weaker than that observed for coeliac 1 Vs control 1 (Table 2). In addition, tests for epistasis using Plink software did not suggest any interaction between these *IL18 *and *IL18RAP *genes in coeliac disease susceptibility.

Haplotype analysis (Table 3) of the *IL18-607/-137 *supported this moderate effect over all haplotypes (P < 0.0001). This is primarily due to the *IL18-607C/-137C *haplotype, and the effect is detectable in the original case control comparison (P = 0.00015, coeliac 1 Vs control 1, Table 3), the follow up sample (P = 0.004, coeliac 2 Vs control 2, Table 3), despite the limited size (N = 171) of the coeliac 2 sample, and in the pooled case-control sample (P < 0.00001, Table 3). Furthermore, the values presented are corrected for the confounding effects of age and sex. The P-values obtained for haplotype analysis are presented without correction for multiple testing, but the global tests (case-control 1, P = 0.00075; case control 2, P = 0.0059; pooled case-control P < 0.00001) are significant after correction for multiple testing across 6 gene regions.

**Table 3:** Haplotype frequencies in Coeliacs and Controls.

IL18-607	IL18-137	*Coeliac	*Control	Hap-Freq	Hap-Score	p-val	sim p-val
**Coeliac 1 Vs Control 1**							
A	C	0.251	0.284	0.277	-0.717	0.473	0.467
A	G	0.136	0.156	0.138	-0.776	0.443	0.438
C	G	0.591	0.558	0.571	0.161	0.872	0.867
C	C	0.022	0.002	0.014	4.056	0.00005	0.00015
							
**Coeliac 2 Vs Control 2**							
A	C	0.173	0.269	0.264	-1.231	0.218	0.241
A	G	0.164	0.127	0.129	1.162	0.245	0.241
C	G	0.591	0.597	0.597	-0.283	0.777	0.789
C	C	0.072	0.007	0.009	3.139	0.002	0.004
							
**Coeliac (1 + 2) Vs Control (1 + 2)**							
A	C	0.234	0.275	0.271	-0.909	0.364	0.364
A	G	0.142	0.138	0.134	0.577	0.564	0.566
C	G	0.591	0.582	0.584	-0.630	0.529	0.531
C	C	0.032	0.005	0.012	4.956	0.00001	0.00001

Case and Control haplotype frequencies were calculated using Phase v2*. All other calculations were performed using Haplostats, and corrected for the effects of age and sex. P values are presented without correction for multiple testing. Haplotype *IL18-607C/-137C *is rare but confers risk (highly significant). Case 1 Vs Control 1, Haplostats global-stat = 16.880, df = 3, P < 0.00075. The same effect is evident when comparing coeliac 2 to control cohort 2, Haplostats global-stat = 12.49, df = 3, P = 0.0059. For comparison of pooled cases (1 + 2) Vs pooled controls (1 + 2), Haplostats global-stat = 25.620, df = 3, P < 0.00001, Phase v2, overall P = 0.01, Hitagene, P < 0.05.

## Discussion

This candidate gene investigation of the chromosome 11 coeliac disease linkage region, which has been identified by several linkage studies [1-3], contains analysis of six genes; *CD3E*, *CD3D*, *CD3G*, *IL10RA*, *THY1 *and *IL18*. Haplotype tagging strategies were used to define haplotypes around the genes of interest, ensuring the maximum information content is gained. Polymorphisms previously shown to be associated with disease or those with a theoretical or proven functional role (e.g. gene promoter regulation or transcription factor binding site alteration) have been included where possible in this study to provide as comprehensive a picture as possible of the genetic variation present in these genes.

Patterns of LD (Supplementary Figure S1, Additional file 1) were generally consistent with those seen in the CEPH European individuals in the Hapmap data, suggesting the genes in question have been effectively tagged. Linkage disequilibrium analysis of the 11q23 region showed that SNPs within each gene showed evidence of LD with other SNPs in the same gene. However, there was little to no inter-gene LD present, except in the *CD3 *gene cluster. Thus the haplotype structure of each gene was assessed separately for association with disease.

The promoter polymorphisms in *IL18 *were initially found to be associated with CD, before correction for multiple testing. Analysis of follow-up samples suggested that this first finding may be due to a haplotypic effect. This is primarily due to the *IL18-607C/-137C *haplotype, and the effect is detectable in the original case control comparison, the follow up sample (despite the limited size [N = 171] of the coeliac 2 sample) and in the pooled case-control sample. Furthermore, the values presented are corrected for the confounding effects of age and sex, and remain significant when considering the effects of multiple testing.

Candidate gene studies may yield conflicting results, which may be due to population stratification, sampling bias, inadequate sample size, variation in study design, and mis-classification of phenotypes [40-42]. Population stratification and sampling bias are unlikely to be an issue, as all cases and controls were unrelated individuals of ethnically uniform Irish origin. Power calculations [43] indicate that our sample sizes afforded > 80% power to detect an effect of genotype relative risk 1.3 for heterozygotes and 1.6 for homozygotes for a range of allele frequencies. While the effect size for *IL18-607 *was higher in the first samples (coeliac 1 Vs control 1, genotype relative risk 1.36 for heterozygotes and 1.84 for homozygotes), the same comparison in the second (much smaller) set of samples was not statistically significant. The combined dataset was consistent with a weak effect for *IL18-607 *(genotype relative risk 1.16 for heterozygotes and 1.33 for homozygotes). However, a much greater effect was observed at the haplotype level, in both case-control sample sets independently and in the combined sample. Using Plink, we estimate the effect size of the *IL18-607C/-137C *haplotype in our combined case-control sample to have an odds ratio = 6.2; thus although rare, it is associated with a relatively large risk. Power calculations using *haplo.power.cc *(Haplostats) suggest that our sample sizes afford > 95% power to detect a haplotypic effect of odds ratio 3 or greater at the 5% level in both our case-control samples, and the combined sample, consistent with our observation of this effect in all three comparisons.

A previous study based on the TDT analysis [29] has examined the relationship between *IL18 *and CD by examination of these two promoter polymorphisms in 105 Spanish families, and found no association with disease. The differences between their result and ours may reflect differences in study design and the rarity of the *IL18-137C/-607C *haplotype, the frequency of which is unknown in Spain. It was not reported by Rueda et al [29] for the Spanish population, but the control frequencies observed by us are similar to those reported for the German and Scottish populations [44].

Four of the 16 SNPs used in this study have been included in a genome-wide analysis of coeliac disease risk [4], where none showed association with disease. While that study did include analysis of *IL18-607 *(rs1946518), *IL18-137 *(rs187238) was not analysed. The haplotype found in this study would not, therefore, have been detected.

Interestingly, follow-up of the top 1500 positive results from coeliac disease whole genome analysis [4] has provided robust replication of association with 7 loci, all of which have a clear functional role in T-cell regulation [35]. Furthermore, based on these findings it has been calculated that non-HLA loci identified to-date contribute approximately 4% to the total CD risk, although this may be an underestimate. Meanwhile, the contribution of HLA-DQ2 and -DQ8 has been calculated at 35% [35]. Therefore, it is apparent that additional factors remain to be discovered. While much of this missing heritability may relate to low risk variants, it is likely that much of the remaining variation may not be well detected by association of single SNPs in the absence of reference to haplotypes.

The observations that all the newly identified risk variants are associated with genes with a known role in a relevant pathway, and that additional risk variants remain to be uncovered, are highly pertinent in the context of directed candidate gene analyses. This approach can be applied to regions of known linkage to disease, and can be focused on pathways (e.g T-cell regulation) that are known to be important in the aetiology of the disease. It is of particular interest that one highly replicated non-HLA CD risk modulator, the *CTLA4 *locus on chromosome 2q33 [45,46] was not detected in linkage studies or the initial genome wide assocation study, although it has since been significantly associated in a large combined type 1 diabetes/coeliac disease study [47]

## Conclusions

Our results suggest a possible haplotypic association between SNPs in the *IL18 *locus and coeliac disease risk. While this may represent a chance statistical departure rather than a true disease association, the same relatively rare haplotype has been noted in two independent rheumatoid arthritis populations [44]. Furthermore, the role of *IL18 *as a CD candidate gene is further strengthened by evidence to suggest that it plays a key role in the maintenance of inflammation in active CD [48]. Directed candidate gene analyses, in combination with more indepth analysis of haplotypic variation may still contribute to our knowledge of complex disease risk, as many relevant effects remain undetected. The 5q and 11q linkage regions contain numerous un-investigated immune-related genes, many of which are plausible candidate genes for CD risk. These may form part of the next phase of candidate gene studies in this condition.

## Abbreviations

CD: coeliac disease; CD3: CD3 antigen; EMA: anti-endomysial antibodies; H4TF-1: H4 gene transcription factor; HLA: human leukocyte antigen; IEL: intraepithelial lymphocytes; IgA: immunoglobulin A; IL10: interleukin 10; IL10RA interleukin 10 receptor A; IL12: interleukin 12; IL18: interleukin 18; IL18RAP; interleukin 18 receptor accessory protein; RA: rheumatoid arthritis; SNP: single nucleotide polymorphism; TDT: Transmission Disequilibrium Test; THY1 (CD90): Thy-1 cell surface antigen; TNFa: tumour necrosis factor alpha; tTG: tissue transglutaminase.

## Competing interests

The authors declare that they have no competing interests.

## Authors' contributions

KB, AWR and RMM designed the study, performed the statistical analysis and drafted the manuscript. KB, AWR, GT and KDP managed sample handling and performed DNA extraction and genotyping. MA, COM, NPK, VT, BE, EC, GL, PMM, FMS, CF and DK evaluated and recruited patients from several centres in Ireland. All authors have read and approved the final manuscript.

## Pre-publication history

The pre-publication history for this paper can be accessed here:

http://www.biomedcentral.com/1471-2350/11/76/prepub

## Supplementary Material

Additional file 1 **Supplementary Figure S1**. Linkage Disequilibrium (D') in the 11q23 region in coeliac cases (a) and controls (b). Click here for file
