# Supplementary material for: Evaluation of 6 candidate genes on chromosome 11q23 for coeliac disease susceptibility: a case control study
Source: BMC Med Genet. 2010 May 17;11:76. doi: 10.1186/1471-2350-11-76 (PMC2880976; doi:10.1186/1471-2350-11-76)
Supplement: Additional file 1 — Supplementary Figure S1. Linkage Disequilibrium (D') in the 11q23 region in coeliac cases (a) and controls (b). [file 1471-2350-11-76-S1.PDF]

a

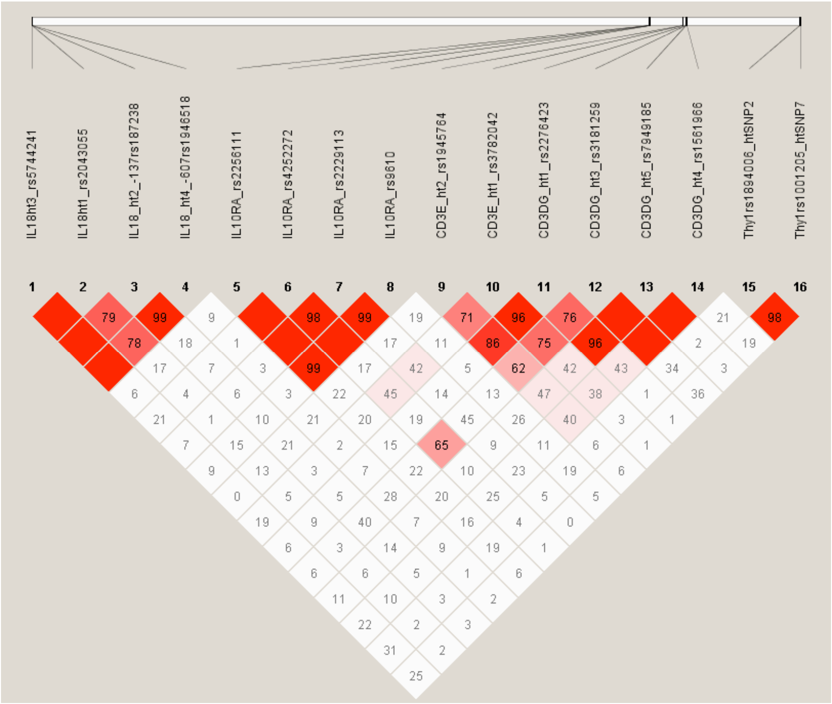

b

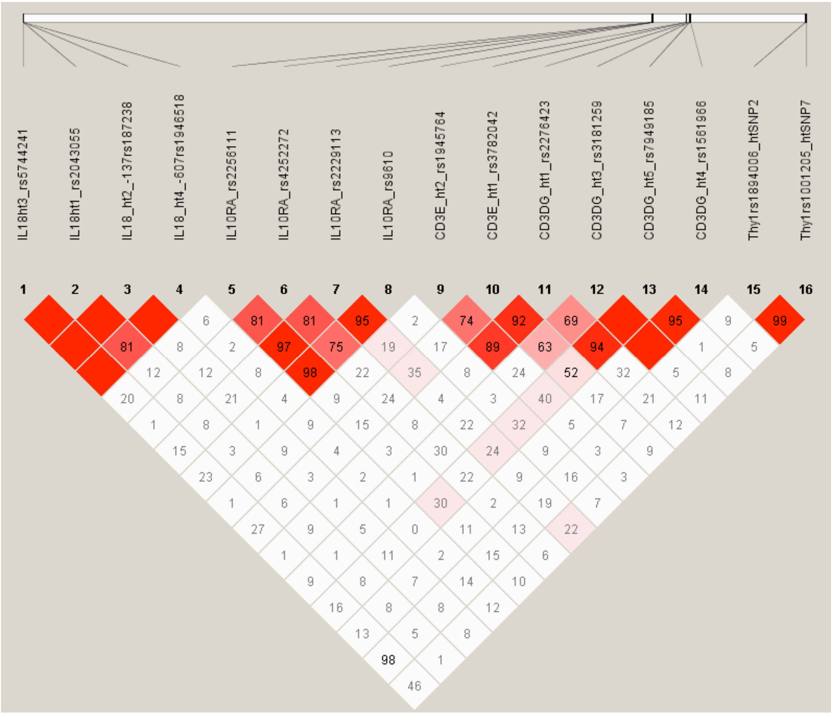

Supplementary Figure 1. Linkage Disequilibrium (D’) in the 11q23 region in coeliac cases (a) and controls (b).
